# Immunometabolic reprogramming and glycolysis-associated signatures in sepsis: insights from single-cell RNA sequencing and machine learning

**DOI:** 10.3389/fimmu.2026.1817391

**Published:** 2026-05-14

**Authors:** Tao Li, Yun Liu, WanZhao Wang, QiuYue Li, Bing Chen

**Affiliations:** 1 Department of Intensive Care Unit, The Second Hospital of Tianjin Medical University, Tianjin, China; 2 Tianjin Institute of Infectious Diseases, The Second Hospital of Tianjin Medical University, Tianjin, China; 3 Department of Emergency Medicine, The Second Hospital of Tianjin Medical University, Tianjin, China; 4 Shaanxi Normal University, Xian, Shaanxi, China; 5 Department of Intensive Care Unit, The First Affiliated Hospital of Zhengzhou University, Zhengzhou, Henan, China

**Keywords:** biomarkers, glycolysis, immunometabolism, machine learning, sepsis, single-cell RNA sequencing (scRNA-seq)

## Abstract

**Background:**

Immunometabolic remodeling is central to sepsis, yet robust glycolysis-associated biomarkers and their cell-type context remain unclear.

**Methods:**

We integrated peripheral blood scRNA-seq (GSE175453) and a whole-blood microarray cohort (GSE100159). Glycolysis activity was scored by AUCell (HALLMARK_GLYCOLYSIS), hub genes were prioritized by LASSO/random forest/Boruta, communication was inferred by ligand–receptor analysis, and qRT-PCR was performed in CLP vs control mice.

**Results:**

Sepsis showed myeloid predominance and increased glycolysis scores, most evident in monocytes and plasma cells. Five candidates (GLRX, MDH1, MDH2, TGFBI, COPB2) displayed good discriminatory performance in bulk data; TGFBI was monocyte-enriched and centrally positioned in a dense communication network with B cells/plasma cells/neutrophils. qRT-PCR confirmed a significant between-group difference for TGFBI in the CLP model.

**Conclusions:**

These findings link enhanced glycolysis-associated programs to a monocyte-centered TGFBI communication pattern and prioritize TGFBI as a candidate biomarker for further validation in sepsis.

## Introduction

Sepsis, as defined by the Sepsis-3 consensus, is characterized as a life-threatening organ dysfunction caused by an uncontrolled host response to infection, with the lungs being one of the most frequently affected organs. When diffuse alveolar-capillary barrier damage leads to hypoxemic respiratory failure, sepsis-associated acute lung injury/acute respiratory distress syndrome (ALI/ARDS) is formed (1). The Global Burden of Disease study indicates that there were approximately 48.9 million cases and 11 million sepsis-related deaths in 2017, suggesting that sepsis remains a significant threat to global public health (2). Sepsis-related ALI/ARDS is associated with an amplification loop involving cytokine storms, complement and coagulation system activation, aberrant activation and migration of macrophages and neutrophils, and impaired alveolar fluid clearance (3, 4). The excessive activation of various inflammatory cells, secretion of inflammatory mediators and cytokines, and dysregulation of redox balance and apoptosis are critical for the progression of ALI (5, 6). Beyond cytokine-driven inflammation, immunothrombosis—the coordinated interaction between innate immune cells, platelets, coagulation pathways, and neutrophil extracellular traps (NETs)—has emerged as an important mechanism linking inflammation to microvascular thrombosis and tissue injury, thereby further aggravating pulmonary vascular permeability and gas-exchange dysfunction in sepsis-associated ALI/ARDS (7, 8).

Activated immune cells undergo metabolic reprogramming to meet the increased energy and biosynthetic demands necessary for proliferation, differentiation, and effector functions (9–11). Among these metabolic adaptations, enhanced glycolysis is a hallmark of inflammatory activation and has been increasingly recognized as a central feature of immune dysregulation in sepsis. Increased glycolytic flux is typically accompanied by lactate accumulation. Beyond being a metabolic byproduct, lactate may also function as a signaling molecule, in part through lactylation, a recently discovered post-translational modification that can regulate protein function and influence biological processes including immune responses and inflammation (12–14). In infection- and sepsis-related settings, accumulating evidence supports a functional role for lactate-associated lactylation. For example, overexpression of HSPA12A reduces glycolysis-derived lactate production, thereby decreasing HMGB1 lactylation and secretion from hepatocytes, suppressing macrophage chemotaxis, and attenuating liver ischemia-reperfusion injury (15). In addition, lactic acidemia has been confirmed to promote the death of alveolar epithelial cells (16). In M1 macrophages under bacterial attack, an intrinsic “lactate clock” has been described, through which lactate-associated histone lactylation may open gene-expression programs related to homeostasis (17). More broadly, glycolysis-derived lactate may couple cellular metabolism to epigenetic regulation through histone lactylation, thereby potentially influencing the transition between inflammatory and reparative programs (18). Consistent with this concept, in sepsis, CD38-high monocytes exhibit hyperactivated glycolysis, leading to activation of hypoxia-inducible factor-1α due to NAD+ consumption (19). Despite the continuous expansion of this mechanistic landscape, the marked biological heterogeneity of patients with acute lung injury/acute respiratory distress syndrome largely explains the inconsistent outcomes of many immune-modulatory interventions in clinical trials (20). These observations suggest that glycolysis-associated immune remodeling may be an important component of sepsis pathobiology.

Despite advances in understanding the pathogenesis of sepsis, current diagnostic and therapeutic strategies remain limited. The biological heterogeneity of patients with Acute Lung Injury (ALI)/Acute Respiratory Distress Syndrome (ARDS), coupled with their complex molecular and immunological mechanisms, underscores the need for new biomarkers and therapeutic targets. Recent studies have highlighted the significance of glycolysis in regulating immune cell function and metabolism, suggesting its potential role in the pathogenesis of sepsis. In this study, we integrated peripheral blood scRNA-seq and microarray data to profile immune-cell composition, glycolysis-associated programs, and intercellular communication in sepsis, and to prioritize candidate genes using machine-learning methods. This analysis provides a framework for understanding immunometabolic remodeling in sepsis and for nominating biomarkers for further validation.

## Materials and methods

### Public dataset acquisition

Two publicly available peripheral blood transcriptomic datasets were obtained from the GEO database. The bulk cohort GSE100159 (GPL6884, Illumina HumanWG-6 v3.0 expression beadchip) comprised 35 sepsis samples and 12 healthy controls and was used for differential expression, immune infiltration, machine-learning–based feature selection, and ROC analysis. The single-cell cohort GSE175453 comprised peripheral immune cells from 4 patients with late sepsis and 5 healthy controls and was generated by CITE-seq from a mixture of whole-blood myeloid-enriched cells and Ficoll-enriched peripheral blood mononuclear cells. This dataset was used for quality control, integration, clustering, glycolysis scoring, and cell–cell communication analysis. Because these datasets differed in disease stage, sample composition, and sequencing platform, they were not merged at the raw-data level; instead, each cohort was used for a distinct analytical purpose to identify concordant glycolysis-associated signals across complementary transcriptomic layers.

### Microarray data preprocessing and differential analysis

Analyses were performed in R (version 4.2.2). Probe annotation was conducted according to the GPL6884 annotation file, and probes were mapped to gene symbols. For genes corresponding to multiple probes, the median expression value was retained. The platform used was the Illumina Human Expression BeadChip. Background correction and quantile normalization were then performed on the Series Matrix data using the normalizeBetweenArrays function in the limma package. Quantile normalization was selected because the bulk cohort was generated from the same Illumina microarray platform, and this approach effectively reduces global between-sample distributional differences while preserving relative expression patterns for downstream differential analysis, machine-learning–based feature selection, and CIBERSORT deconvolution. Low-expression probes were excluded if at least 20% of samples showed zero or background-level expression. Differential expression analysis between the sepsis and control groups was conducted using a linear model implemented in the limma package, and multiple testing was adjusted using the Benjamini–Hochberg method. Genes with a false discovery rate < 0.05 and an absolute log2 fold change > 0.25 were considered significantly differentially expressed. Volcano plots, scatter plots, and bar plots were generated using ggplot2.

### Immune infiltration assessment

The proportions of 22 immune-cell types were estimated using CIBERSORT with the LM22 signature and 1,000 permutations. Quantile normalization (QN = TRUE) was applied to the microarray data to match the assumptions of the algorithm. Only samples with a CIBERSORT p-value < 0.05 were retained for downstream statistical analysis and visualization.

### Single-cell data processing and integration

Using Seurat v4, we computed nFeature_RNA, nCount_RNA, the percentage of mitochondrial transcripts (pMT), and the percentage of hemoglobin gene transcripts (pHB) for each sample. Cells were retained if nFeature_RNA was between 200 and 5,000, nCount_RNA was between 200 and 30,000, pMT was <30%, and pHB was <5%. These thresholds were selected based on the QC distributions of the public dataset and commonly used filtering principles for blood-derived scRNA-seq data. The relatively permissive mitochondrial cutoff was used to avoid excessive exclusion of biologically relevant but stress-associated immune cells in the late-sepsis cohort, while the additional hemoglobin filter helped reduce contamination by erythrocyte-rich droplets. Gene expression was normalized with the LogNormalize method (scale.factor = 1e4), followed by PCA for dimensionality reduction. Given the small sample size and between-sample variability in the public late-sepsis cohort, Harmony was applied after quality control to reduce sample-level technical variation before downstream clustering and visualization. Batch-correction performance was quantitatively assessed using the integration local inverse Simpson’s index (iLISI). To avoid confounding by true disease-related transcriptional differences, iLISI was calculated separately within the Control and Sepsis groups using sample identity (orig.ident) as the batch label. A shared nearest neighbor (SNN) graph was constructed based on the first 15 Harmony-corrected dimensions, and cells were clustered using the Louvain algorithm at a resolution of 1.0. UMAP was used for visualization in the integrated low-dimensional space. Cell types were manually annotated and cross-validated based on canonical marker genes (B cells: MS4A1, CD79A/B; T cells: CD3D/E, TRAC; NK cells: NKG7, GNLY, PRF1; neutrophils: S100A8/A9, LTF, ELANE, MPO; monocytes: LYZ, FCGR3A; platelets: PPBP, PF4; plasma cells: JCHAIN, MZB1, etc.), and the annotations were visualized using dot plots and violin plots. Cluster identities were assigned only when multiple canonical markers showed concordant enrichment within the same cluster, and the annotations were cross-validated by UMAP distribution, dot plots, and violin plots.

### Genetic set scoring and inter-group comparison

Because glycolysis represents a central metabolic program underlying lactate accumulation and immunometabolic remodeling in sepsis, we selected the curated HALLMARK_GLYCOLYSIS gene set from MSigDB as a transcriptomic proxy for glycolysis-associated activity at the single-cell level. AUCell activity scoring was then performed using HALLMARK_GLYCOLYSIS (MSigDB) at the single-cell level; AUCell scores were compared between the control and sepsis groups in the overall cell population and within each cell type using two-sided Wilcoxon rank-sum tests with Benjamini-Hochberg correction. Additionally, Spearman correlation analysis between AUCell scores and whole-genome expression was performed at the single-cell level to identify top-correlated genes for subsequent presentation and enrichment analysis.

### Differential genes and enrichment based on AUCell grouping

Cells were dichotomized into score_UP and score_DOWN groups using the median AUCell score as the cutoff. Differentially expressed genes were identified using Seurat::FindMarkers (Wilcoxon rank-sum test), with thresholds of FDR < 0.05 and |avg_log2FC| > 0.25. Functional enrichment was performed using clusterProfiler. For KEGG analysis, enricher was applied with a customized TERM2GENE/TERM2NAME annotation. GO enrichment (BP/MF/CC) was conducted using enrichGO (Org.Hs.eg.db). Reactome enrichment was performed using enrichPathway (ReactomePA). For GSEA, all genes in the microarray were ranked according to their association/correlation or effect size with the target variable, and GSEA was performed with minGSSize = 10, maxGSSize = 500, nPerm = 10,000, and Benjamini–Hochberg (BH) adjustment. For single-gene GSEA (TGFBI), to delineate TGFBI-associated pathways, the Spearman correlation coefficient between each gene and TGFBI expression was computed across microarray samples; genes were then ranked by this correlation and KEGG/Reactome GSEA was performed. Enrichment curves and NES/Padj dot plots were generated for summary visualization.

### Cell communication analysis

Based on the single-cell annotation matrix, cell-to-cell communication was inferred using CellChat (CellChatDB.human). An expression matrix and corresponding metadata object were first constructed. The probability and significance of ligand-receptor interactions were then calculated using permutation testing (p < 0.05). Communication frequency networks (counts) and the interaction strength networks (weights) were subsequently generated. Chord plots were constructed focusing on B cells, neutrophils, plasma cells, TGFBI-Monocytic cells, and TGFBI+Monocytic cells. Major signaling axes were summarized, and bubble plots were generated from both source and target perspectives.

### Feature gene selection and model evaluation

Feature selection was conducted using the microarray expression matrix. LASSO analysis was performed with the glmnet package using binary logistic regression and 10-fold cross-validation, with lambda.min and lambda.1se used as references. Random forest analysis was performed using the randomForest package (ntree = 1000), and MeanDecreaseGini was extracted as the variable-importance metric. Boruta analysis was performed using the Boruta package (maxRuns=100) to identify stable features. The intersection of the three methods was used to define the candidate gene set. The diagnostic performance of individual genes in distinguishing sepsis from controls was evaluated using the area under the receiver operating characteristic curve (AUC) with the pROC package.

### qRT-PCR analysis

According to the manufacturer’s instructions, RNA was extracted from the white blood cell pellets of C57BL mice (sepsis group and healthy control group, n=6) using an RNA isolation kit (Beyotime, R00240). The total RNA was subsequently treated with DNaseI to digest genomic DNA. The quality and quantity of the RNA were determined by gel electrophoresis and spectrophotometry. After treating the samples with RNase inhibitor, RNA was reverse-transcribed into cDNA using the All-In-One 5X RT Master Mix kit (ABM, G592). Finally, real-time quantitative reverse transcription polymerase chain reaction (qRT-PCR) analysis was performed on the Rotor-GeneQ instrument (QIAGEN, Germany) using BlasTaq™ 2X qPCR Master Mix (ABM, G891). The qRT-PCR conditions were as follows: pre-denaturation at 95 °C for 10 minutes, followed by 40 cycles of denaturation at 95 °C for 10 seconds, annealing at 61 °C for 20 seconds, and extension at 72 °C for 25 seconds. α-Tubulin was used as the internal control.

### Statistics and visualization

Unless otherwise specified, continuous variables were compared using the two-sided Wilcoxon rank-sum test or Spearman correlation analysis. Multiple testing was corrected using the Benjamini–Hochberg method (p.adjust), and statistical significance was defined as FDR < 0.05. The ggplot2, Seurat, pheatmap, ComplexHeatmap, circlize, and CellChat packages were used for visualization. All analyses were performed in R (v4.2.2), and reproducible scripts can be provided upon request. For qRT-PCR, gene expression was normalized to α-Tubulin as the internal control, and relative expression levels were calculated using the 2^−ΔΔCt method. Data were presented as mean ± SEM. Two-sided Welch’s t-test was used for group comparisons. *p < 0.05; ns, not significant.

## Results

### Data quality control and integration

**Figure 1:**
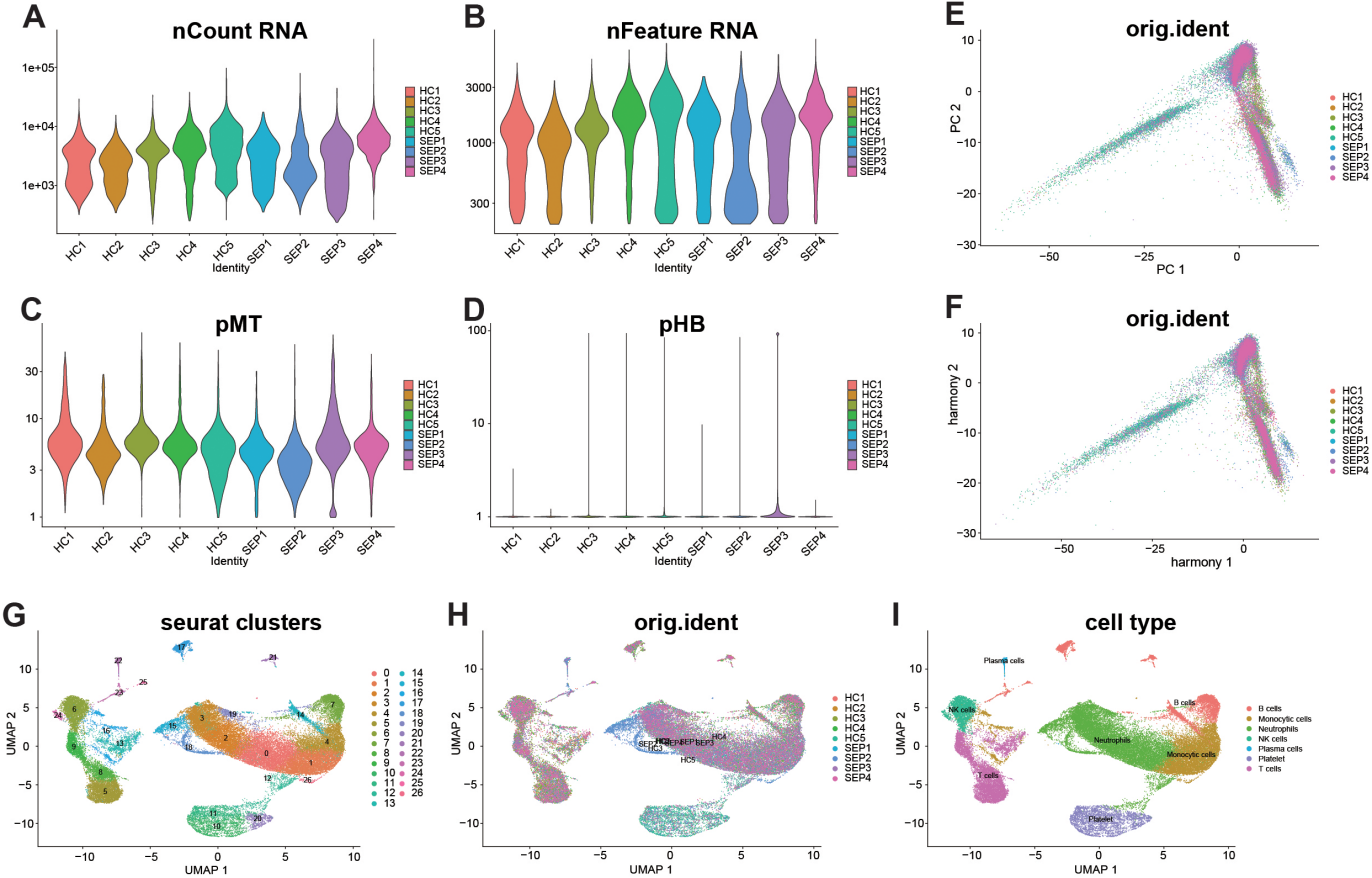
Overview of sample-level QC and integration. **(A–D)** Quality control was performed based on nFeature_RNA (200–5,000), nCount_RNA (200–30,000), pMT (<30%), and pHB (<5%). **(E)** PCA shows batch effects. **(F)** After Harmony correction (dims = 1–15), samples are well mixed. **(G, H)** UMAP visualization of clusters and sample distribution. A total of 6,635,520 cell barcodes were included, and 46,710 cells were retained after QC. **(I)** UMAP annotated by cell type.

At the cellular level, we implemented rigorous quality control (QC): retaining cells with 200–5,000 genes, 200–30,000 unique molecular identifiers (UMIs), mitochondrial fraction <30%, and hemoglobin fraction <5% (**Figures 1A–D**). Initial principal component analysis (PCA) revealed significant batch/individual differences (**Figure 1E**); after integration using Harmony (dims = 1–15), samples were adequately mixed in the low-dimensional space, with batch effects markedly reduced (**Figure 1F**). Quantitative batch-mixing evaluation further supported improved sample integration after Harmony, with the mean iLISI increasing from 1.57 to 2.45 in the Control group and from 1.21 to 2.17 in the Sepsis group. Following clustering and visualization with UMAP in the integrated space, a stable cellular cluster structure with good cross-sample consistency was obtained (**Figures 1G, H**). Based on classical marker genes, seven major immune/blood cell clusters were identified: B cells, monocytes, neutrophils, natural killer (NK) cells, plasma cells, platelets, and T cells (**Figure 1I**). Overall, 46,710 high-quality cells were retained from the initial 6,635,520 raw cell barcodes for downstream analysis. The marked reduction after QC mainly reflected the removal of low-quality barcodes/droplets, barcodes with excessive mitochondrial or hemoglobin signals, and noninformative events unlikely to represent intact cells.

### Cell type annotation and inter-group compositional differences

**Figure 2:**
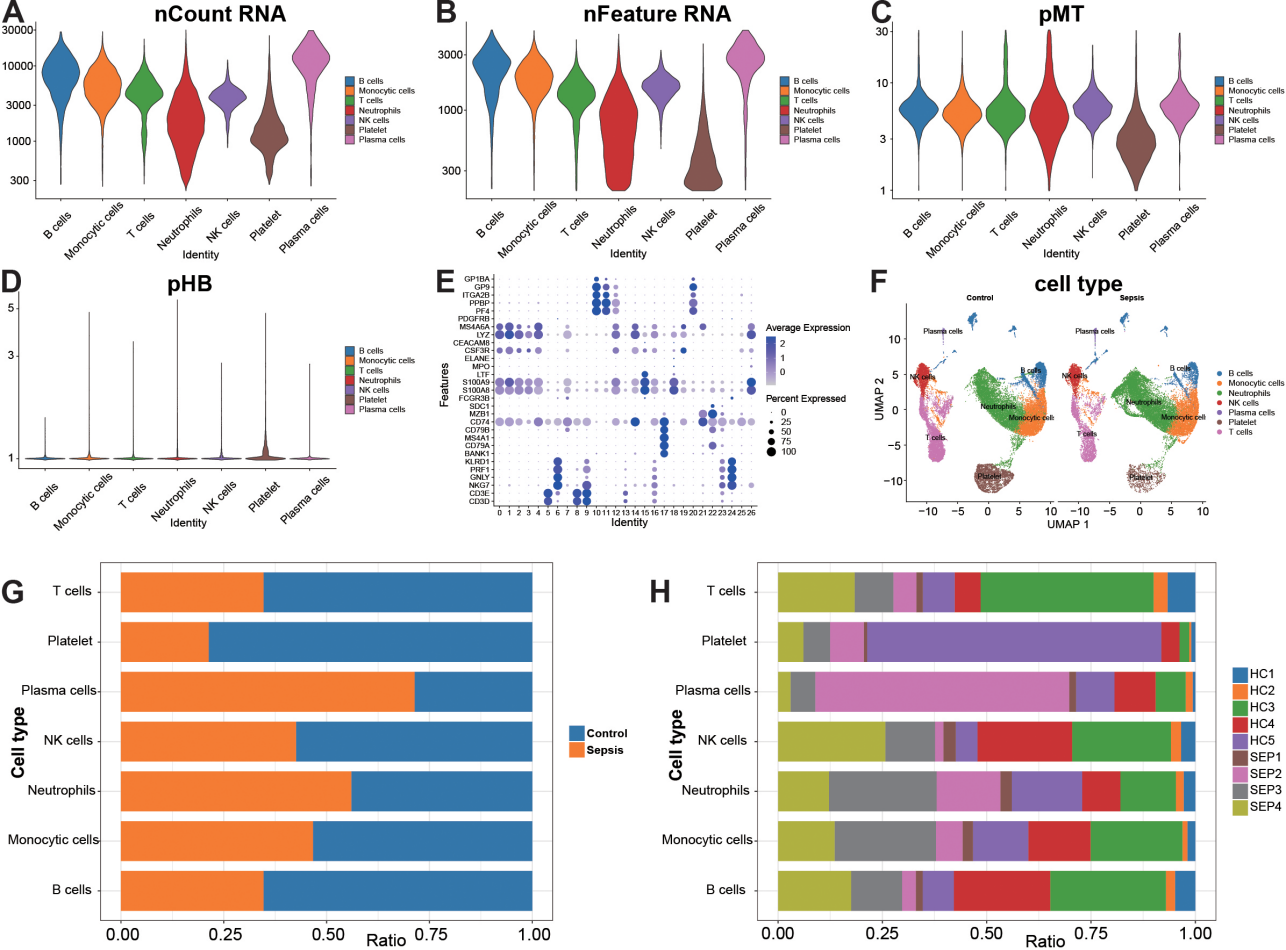
Cell type annotation and composition. **(A–D)** Distributions of nCount_RNA, nFeature_RNA, pMT, and pHB across different cell types. **(E)** Mean expression levels of the annotation marker genes and the proportion of cells expressing each marker. **(F)** UMAP colored by cell type and faceted by Control/Sepsis. **(G, H)** Cell-type proportions summarized at the group level and at the individual sample level.

The distributions of nCount_RNA, nFeature_RNA, pMT, and pHB across various cell types are generally reasonable, with data quality sufficient to support subsequent analyses (**Figures 2A–D**). The typical marker genes are consistent with the annotations: MS4A1/CD79A marks B cells, CD3D/E marks T cells, NKG7/PRF1 marks NK cells, LYZ/S100A8-A9 marks monocytes/neutrophils, PF4/PPBP marks platelets, and MZB1/SDC1 marks plasma cells (**Figure 2E**). A UMAP plot colored by cell type and stratified by Control/Sepsis groups shows similar distributions between the two groups, with batch correction adequately performed. Additionally, there is a trend indicating relative expansion of innate immune clusters (monocytes/neutrophils) in sepsis samples (**Figure 2F**). Comparative composition analysis at the group level reveals an increased proportion of monocytes and neutrophils in the sepsis group, whereas the proportions of B/T cells, NK cells, and platelets are reduced. Plasma cells show a relative increase at the group level (**Figure 2G**). Stacked bar plots at the individual level further demonstrate that the aforementioned trends are predominantly observed across most samples but with varying magnitudes, suggesting biological heterogeneity (**Figure 2H**).

### Single-cell AUCell profiling of glycolysis-associated activity in sepsis

**Figure 3:**
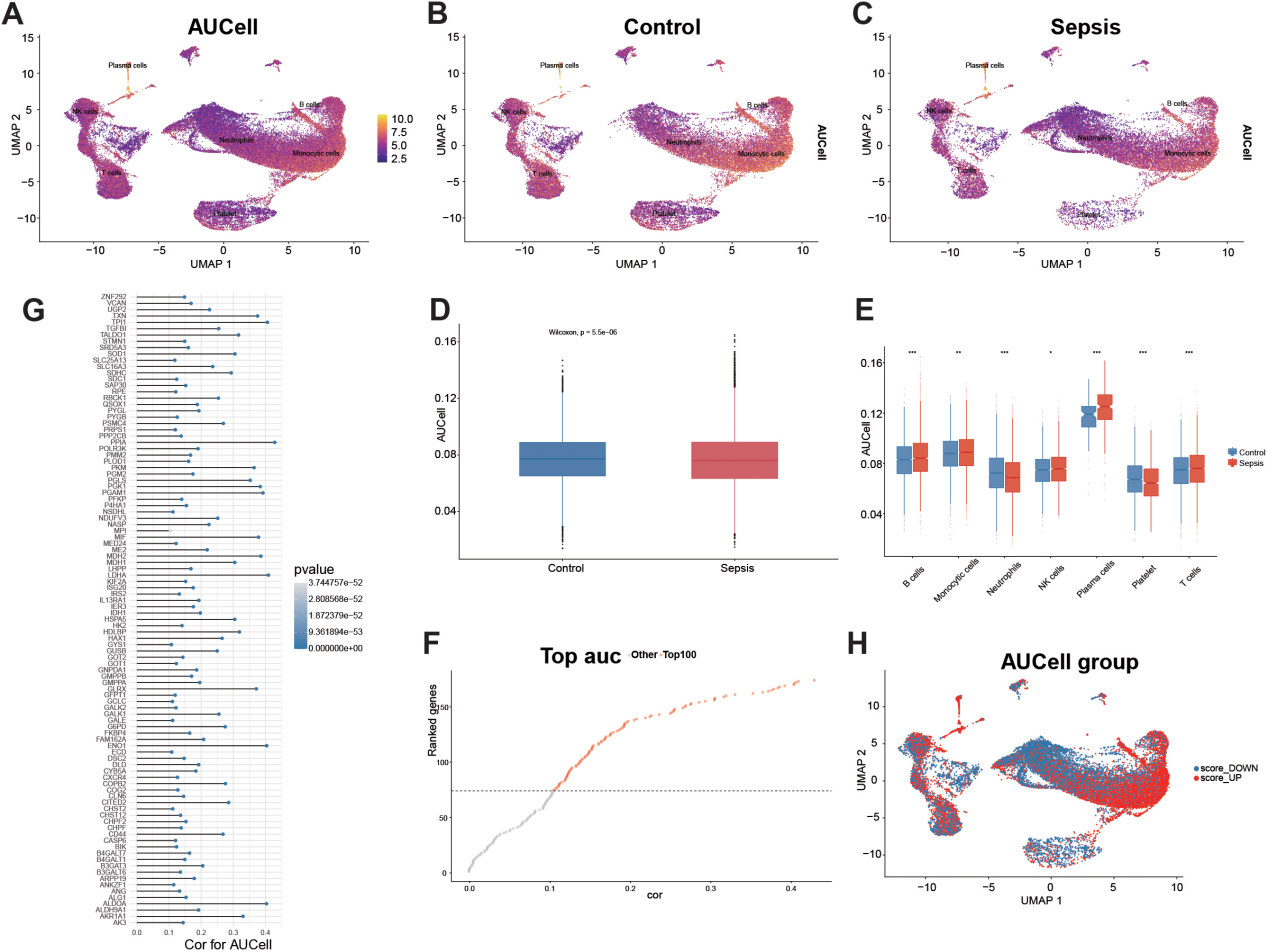
Glycolytic activity predicted by AUCell. **(A)** UMAP showing AUCell scores calculated from the HALLMARK_GLYCOLYSIS gene set. **(B, C)** AUCell UMAPs stratified by Control and Sepsis groups. **(D)** Comparison of AUCell score distributions between groups for all cells (center line: median; box: IQR; whiskers: 1.5×IQR; two-sided Wilcoxon rank-sum test). **(E)** AUCell differences between groups within each major cell type (each dot represents a single cell; significance indicated as *, **, *** for p < 0.05, 0.01, 0.001; Wilcoxon test). **(F)** Ranked plot of Spearman correlations between individual genes and AUCell scores. **(G)** Correlation coefficients and multiple-testing–adjusted p values (FDR) for the top AUCell-correlated genes. **(H)** Cells were divided into score_UP and score_DOWN using the median AUCell score, and their spatial distributions were visualized on the UMAP.

Based on the HALLMARK_GLYCOLYSIS gene set, the AUCell activity showed heterogeneous distribution across the whole graph, with significantly stronger signals in monocyte regions and relatively weaker signals in platelet and lymphocyte regions (**Figure 3A**). After stratification, it was found that sepsis samples exhibited higher AUCell intensity in a large number of cellular regions, with the majority of the differences concentrated around monocytes/plasma cells (**Figures 3B, C**). Overall comparison revealed a significantly elevated AUCell in sepsis (**Figure 3D**). When stratified by cell type, the AUCell of monocytes and plasma cells was significantly increased in sepsis, while that of neutrophils, T cells, NK cells, and platelets was decreased; B cells showed a smaller change but still had a statistically significant difference (**Figure 3E**). Correlation analysis between individual genes and AUCell scores showed that the top-ranked genes reached Spearman correlation coefficients of approximately 0.4, suggesting coordinated expression with glycolysis-associated transcriptional activity (**Figures 3F, G**). When the AUCell median was used as a threshold to divide cells into score_UP and score_DOWN groups, score_UP cells were mainly aggregated in monocytes and some plasma cells, while score_DOWN cells were more commonly found in platelets and lymphocytes (**Figure 3H**).

### Differential expression and functional enrichment associated with high glycolysis AUCell scores

**Figure 4:**
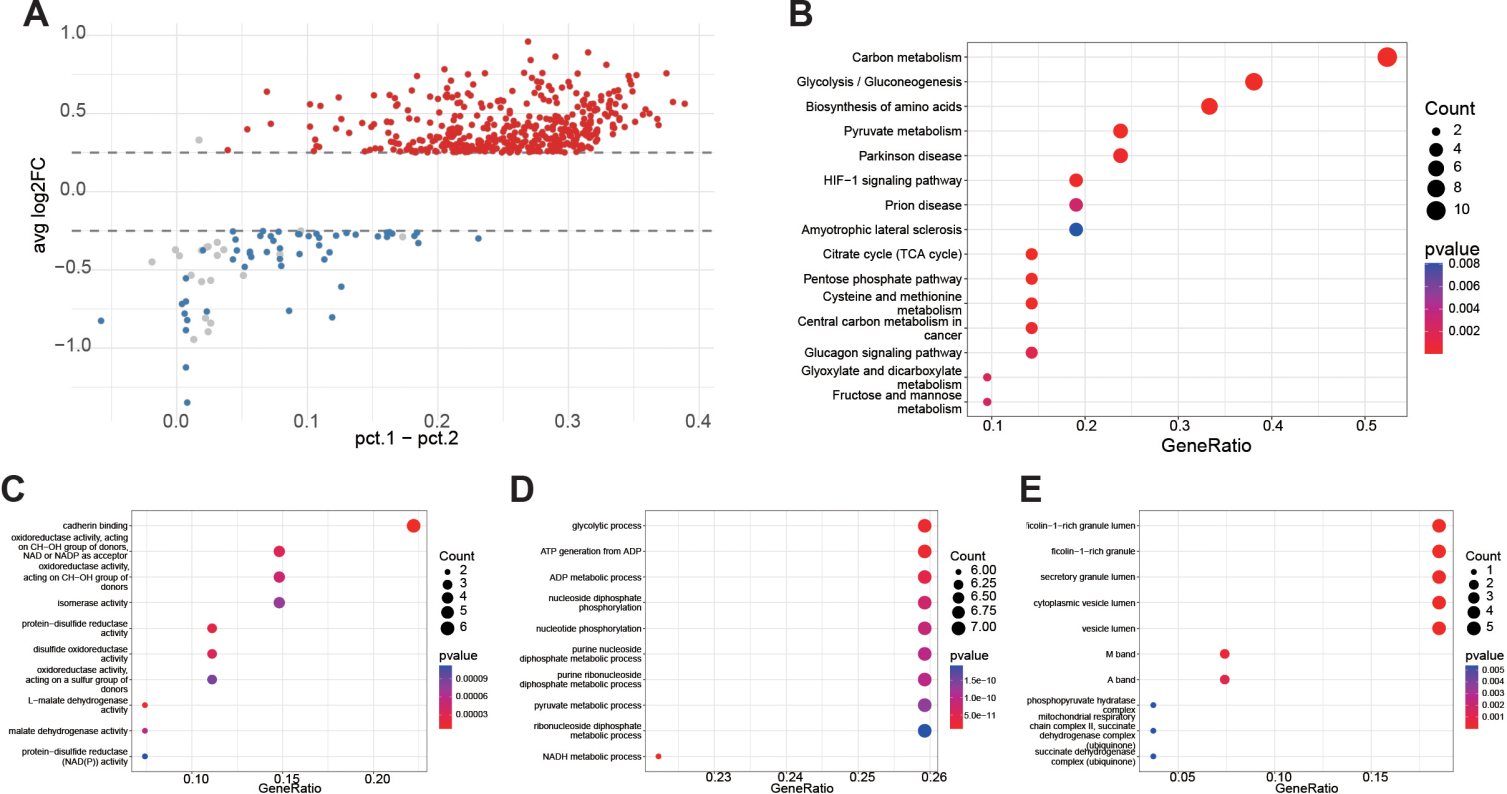
Differential expression and pathway enrichment with glycolysis-related AUCell scores. **(A)** Differential gene scatter plot (score_UP vs score_DOWN). The x-axis indicates the difference in the percentage of expressing cells (pct.1 − pct.2), and the y-axis indicates the average log fold change (avg_log2FC). Red: upregulated; blue: downregulated; gray: not significant (threshold: FDR < 0.05). Upregulated genes in score_UP cells generally show a higher fraction of expressing cells than in score_DOWN cells. **(B)** KEGG enrichment (enricher, customized TERM2GENE/TERM2NAME). Top pathways are mainly enriched in Carbon metabolism, Glycolysis/Gluconeogenesis, Biosynthesis of amino acids, and Pyruvate metabolism. Bubble size represents gene counts, and color indicates P values. **(C)** GO Molecular Function (GO-MF) enrichment. Significant terms include oxidoreductase activity, protein-disulfide reductase activity, and malate dehydrogenase activity, highlighting redox-related functions. **(D)** GO Biological Process (GO-BP) enrichment. Significant terms are mainly related to glycolytic process, nucleotide/ADP/ATP metabolism, and pyruvate metabolic process, reflecting energy metabolism and phosphorylation-associated processes. **(E)** GO Cellular Component (GO-CC) enrichment. Terms suggest enrichment in granule lumen and mitochondrial respiratory chain complexes, further supporting an organelle basis for metabolic pathways.

After dichotomizing cells into score_UP and score_DOWN groups using the median AUCell scores, differential expression analysis identified genes upregulated in score_UP cells relative to score_DOWN cells (FDR < 0.05). These upregulated genes were also detected in a higher fraction of score_UP cells (**Figure 4A**). KEGG enrichment analysis revealed significant entries primarily concentrated in Carbon metabolism, Glycolysis/Gluconeogenesis, Biosynthesis of amino acids, and Pyruvate metabolism (**Figure 4B**). GO analysis further indicated enrichment in redox-related activities at the molecular function level (**Figure 4C**), with biological processes predominantly focused on glycolysis and nucleotide/energy generation processes (**Figure 4D**). At the cellular component level, enrichment was observed in the localization of peroxisomal cavities and mitochondrial respiratory chain complexes, supporting the organelle-based foundation of metabolic pathways (**Figure 4E**).

### Machine-learning-based prioritization of glycolysis-associated candidate genes and their association with immune infiltration

**Figure 5:**
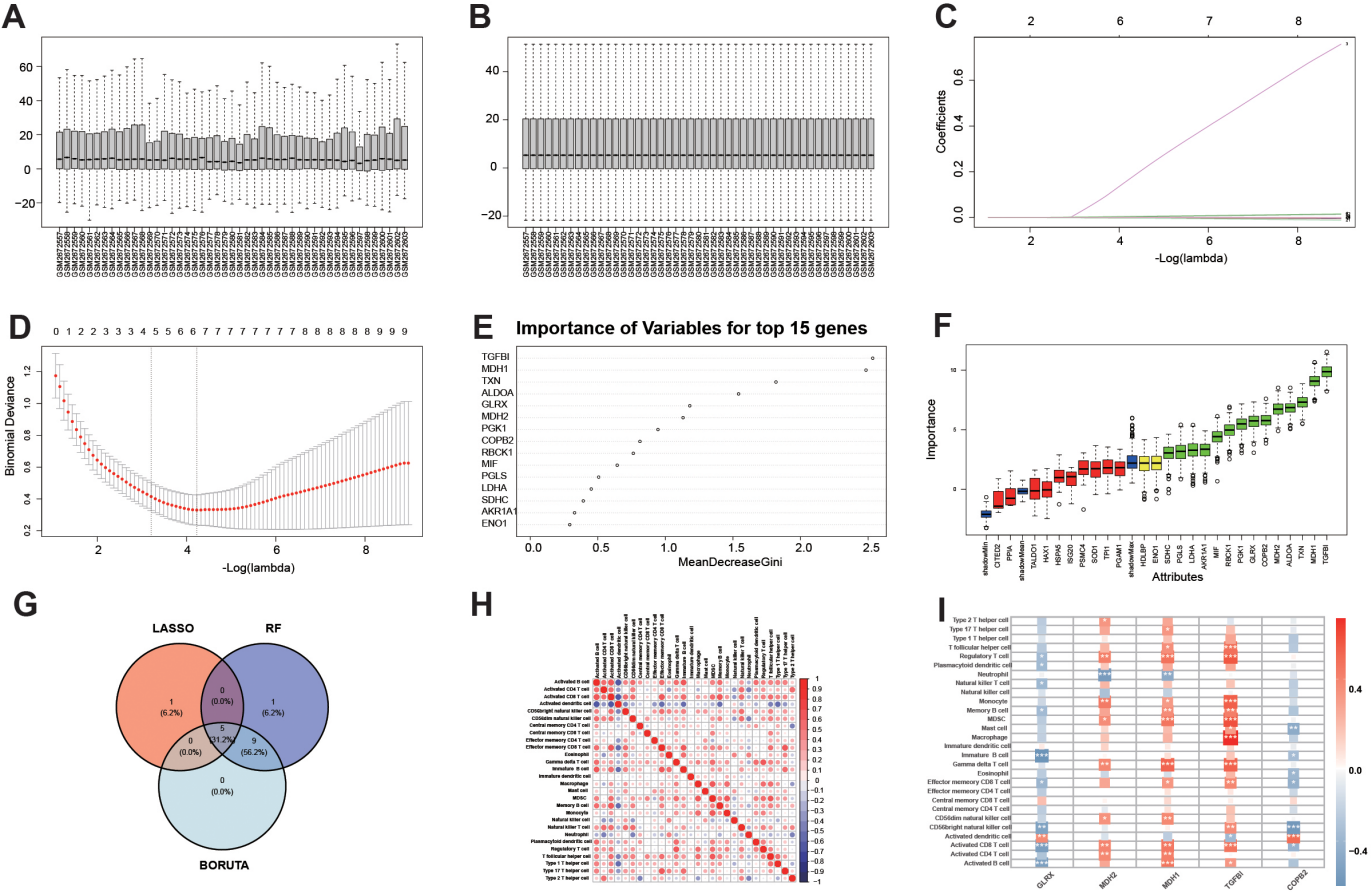
Gene selection workflow and association with immune infiltration. **(A)** Overview of pairwise correlations of whole-transcriptome expression among samples, showing that the overall distribution and batch consistency were suboptimal. **(B)** Expression distribution across samples after quantile normalization using limma::normalizeBetweenArrays; medians were aligned and dispersion became more comparable. **(C)** LASSO coefficient path plot. The x-axis indicates −log(λ) and the y-axis indicates regression coefficients, illustrating coefficient shrinkage and the set of selected genes as the penalty changes. **(D)** Cross-validation curve for LASSO showing the mean ± SE of binomial deviance across λ values; vertical dashed lines indicate λ_min and λ_1SE, which were used to determine the optimal model. **(E)** Random forest variable importance plot showing the top 15 genes ranked by importance (MeanDecreaseGini). **(F)** Boruta feature selection: boxplots of feature importance for the final confirmed genes. **(G)** Venn diagram showing the intersection of genes selected by the three methods (LASSO/RF/Boruta). **(H)** Correlation heatmap of immune cell proportions inferred by CIBERSORT, with significance annotations. **(I)** Correlation heatmap between candidate hub genes and immune cell types; colors indicate correlation coefficients (red, positive; blue, negative), and asterisks denote significance (P < 0.05, *P < 0.01, **P < 0.001).

To connect the glycolysis-associated transcriptional programs identified at the single-cell level with bulk transcriptomic biomarkers, we next prioritized metabolism-related candidate genes from the microarray cohort using complementary machine-learning algorithms. Significant variations and uneven correlations were observed in the original expression distributions of various samples (**Figure 5A**). To reduce non-biological between-sample distributional differences in the Illumina microarray cohort, we applied quantile normalization using limma::normalizeBetweenArrays. After normalization, the median values were aligned, and the dispersion significantly converged, markedly enhancing the comparability among samples (**Figure 5B**). On this basis, the coefficient path of LASSO showed that only a few genes entered the model as the penalty increased, with coefficients progressively shrinking (**Figure 5C**). The cross-validation curve determined a sparse and robust set of candidate genes based on λ_min/λ_1SE (**Figure 5D**). In terms of variable importance provided by the random forest, TGFBI ranked at the forefront, followed by energy metabolism-related genes such as MDH1/MDH2, TXN, and ALDOA (**Figure 5E**). Boruta further stabilized and confirmed a set of “important” features through multiple permutations (**Figure 5F**). The intersection of these three algorithms yielded five robust candidate genes: GLRX, MDH1, MDH2, TGFBI, and COPB2 (**Figure 5G**). Although these genes are not all canonical glycolytic enzymes, they are linked to glycolysis-associated metabolic remodeling through redox regulation (GLRX), malate shuttle and NAD(H) balance (MDH1/MDH2), extracellular-microenvironmental signaling (TGFBI), and vesicular/secretory transport under metabolically activated states (COPB2). The inferred proportions of immune cells by CIBERSORT exhibited typical correlation structures, suggesting a collaborative and antagonistic pattern between innate and adaptive immune systems (**Figure 5H**). At the gene-immune cell correlation level, TGFBI showed a significant positive correlation with monocytes and other innate immune cells, whereas COPB2 was predominantly negatively correlated with various T cell subsets. Additionally, MDH1/MDH2 displayed consistent directional associations with multiple immune cell types, supporting their roles in the remodeling of the immune microenvironment (**Figure 5I**).

### Expression alterations of metabolism-related candidate genes in sepsis and their diagnostic significance

**Figure 6:**
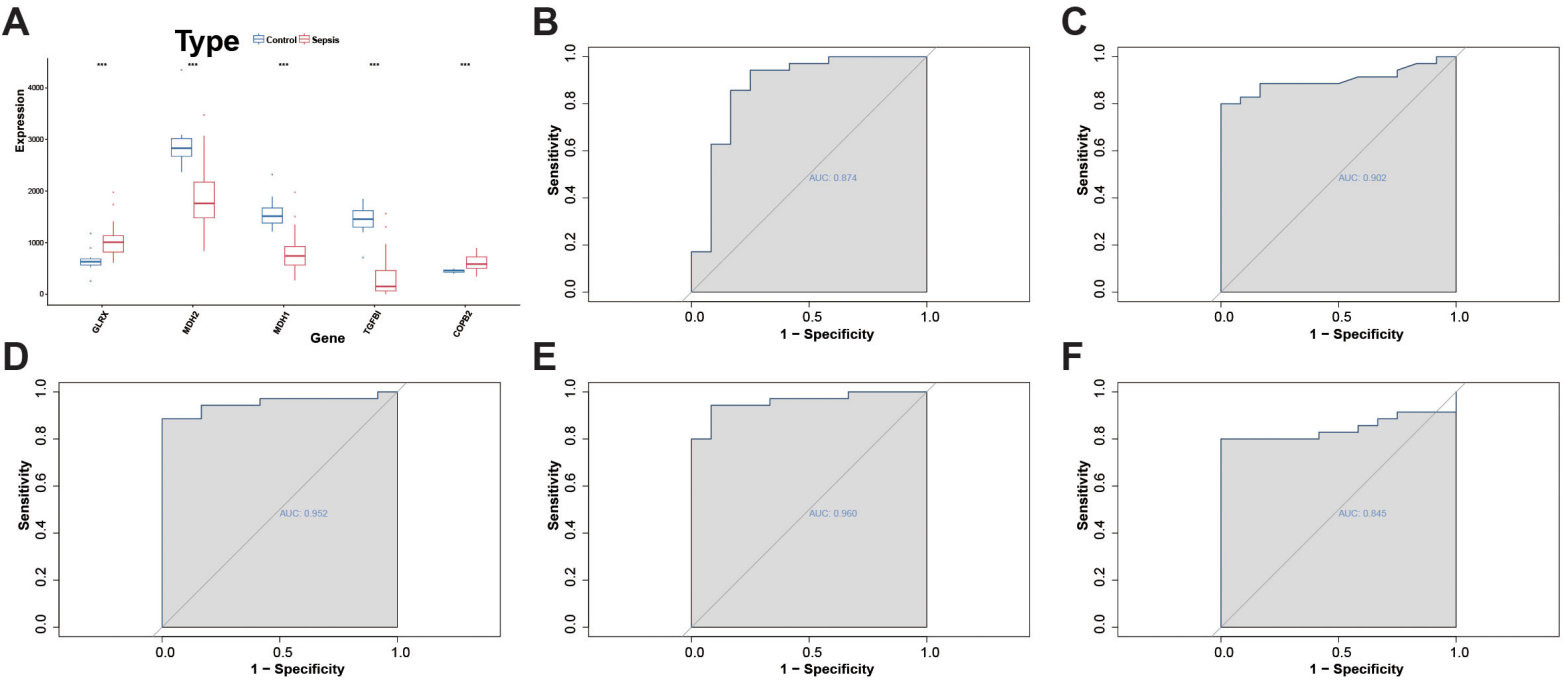
Expression differences and diagnostic performance of metabolism-related candidate genes in sepsis. **(A)** Boxplots showing the expression differences of the five feature genes between the Control and Sepsis groups. **(B–F)** ROC curves and corresponding AUC values for each of the five genes, evaluating their ability to discriminate Sepsis from Control samples.

In the peripheral blood transcriptome, we compared the expression of five metabolic-related candidate genes between sepsis and control samples. GLRX and COPB2 were significantly upregulated in sepsis, whereas MDH1, MDH2, and TGFBI were significantly downregulated, with all comparisons showing statistical significance (**Figure 6A**). Univariate ROC analysis revealed that TGFBI exhibited the highest discriminative power (AUC = 0.960), followed by MDH1 (AUC = 0.952) and MDH2 (AUC = 0.902). GLRX (AUC = 0.874) and COPB2 (AUC = 0.845) also demonstrated certain diagnostic efficacy (**Figures 6B–F**). These findings suggest that these five gene features may have potential applications in the classification and screening of sepsis.

### Genome-wide GSEA and single-cell expression distribution of TGFBI

**Figure 7:**
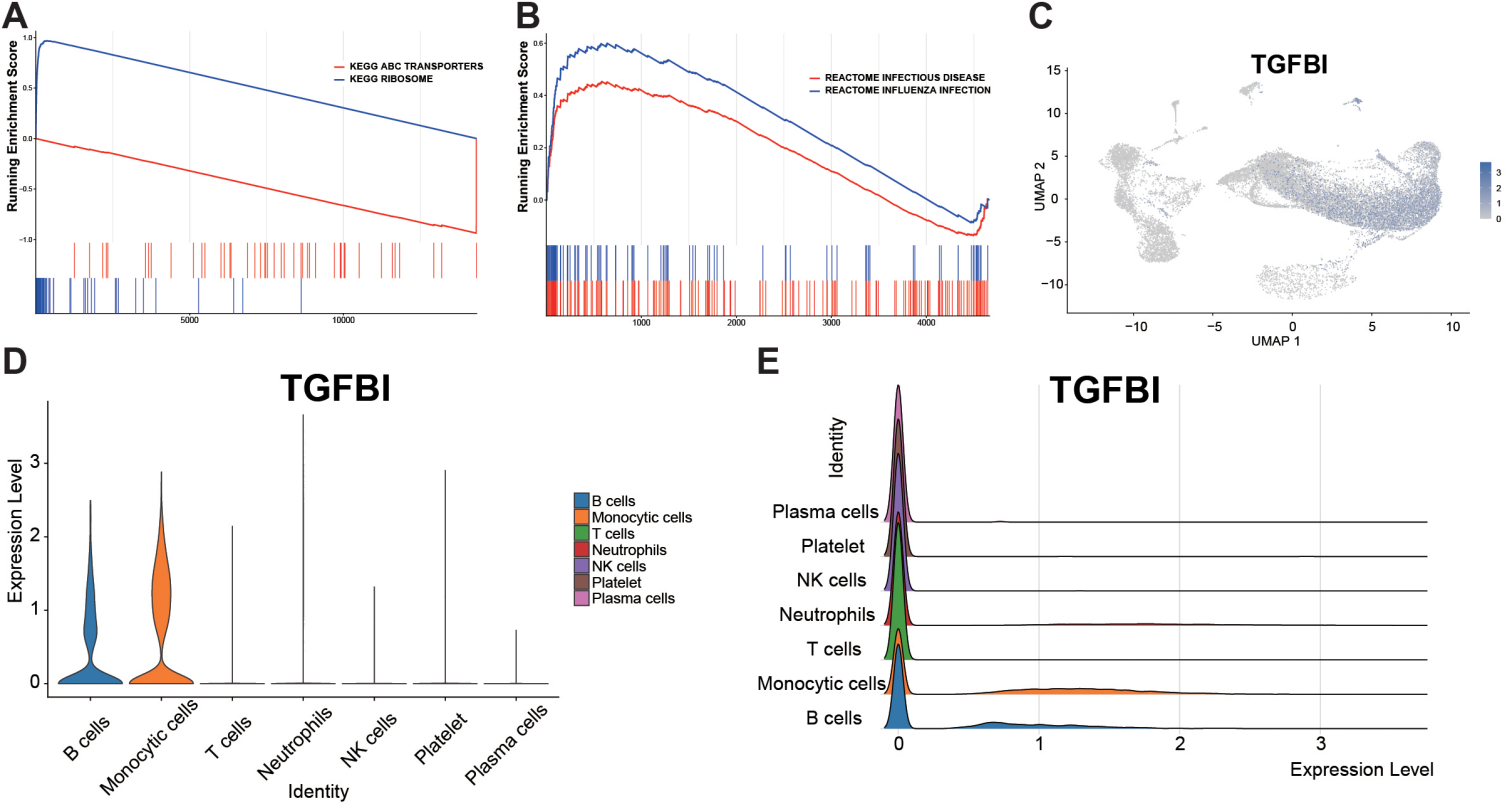
GSEA and single-cell expression characteristics of TGFBI. **(A)** GSEA based on a genome-wide ranked list (KEGG). The Ribosome pathway shows positive enrichment at the leading edge of the ranked list, whereas ABC transporters shows negative enrichment toward the bottom of the ranked list. **(B)** Reactome-based GSEA. The Influenza infection and Infectious disease pathways show positive enrichment at the leading edge of the ranked list. **(C)** Spatial expression distribution of TGFBI across the peripheral immune cell atlas (UMAP). **(D)** Violin plots of TGFBI expression across major cell types: Monocytes show markedly high expression, B cells show moderate expression, and other cell types show low expression. **(E)** Ridge plots of TGFBI expression across cell types: further indicating that monocytes exhibit the highest expression peak, while other cell types are concentrated in the low-expression range.

Based on the whole-genome sequencing GSEA (KEGG), the Ribosome pathway was significantly positively enriched, while the ABC transporters pathway was negatively enriched, indicating an enhancement of protein synthesis programs and a decrease in transmembrane transport activity (**Figure 7A**). The GSEA of the Reactome database showed significant positive enrichment of pathways related to immune/infection, such as Influenza infection and Infectious disease (**Figure 7B**). At the single-cell level, the spatial distribution of TGFBI was mainly localized in the monocyte cluster, with weaker signals in other cell types (**Figure 7C**). The violin plots and ridge plots consistently confirmed that monocytes had the highest expression, followed by moderate expression in B cells, and low expression in other cell types (**Figures 7D, E**).

### Cell communication networks and key signaling axes

**Figure 8:**
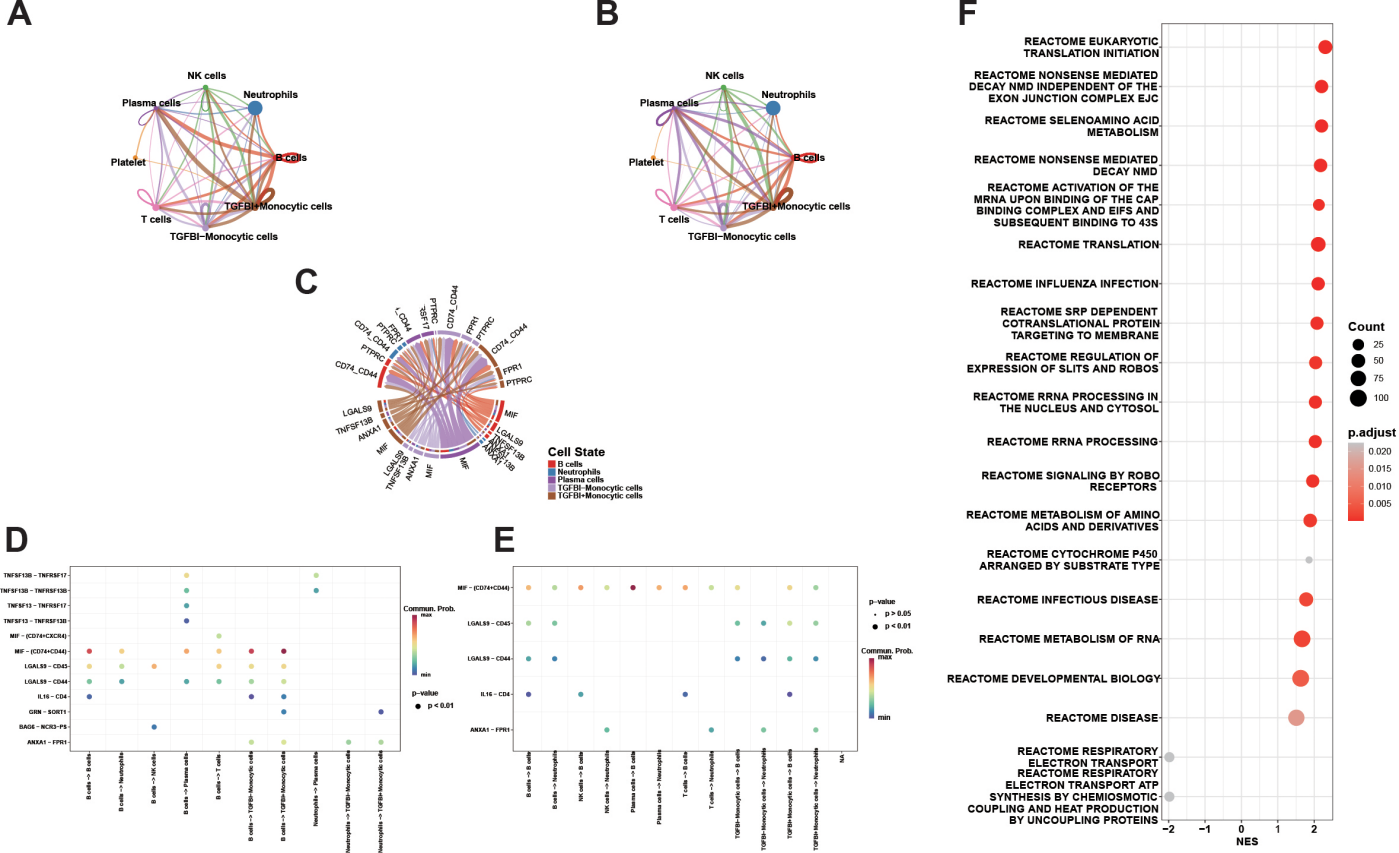
Overview of cell–cell communication and pathway enrichment analysis. **(A)** Interaction number network (Counts). The number of inferred cell–cell communications based on ligand–receptor pairs. Nodes represent cell types; edge thickness reflects the number of significant interactions between two cell groups, and node size is proportional to the total number of interactions involving that cell type. **(B)** Interaction strength network (Weights). Using the same cell-type network, edges are weighted by the inferred communication strength/probability; thicker edges indicate stronger overall communication. **(C)** Representative ligand–receptor chord diagram. High-confidence interactions among B cells, Neutrophils, Plasma cells, TGFBI− Monocytic cells, and TGFBI+ Monocytic cells are summarized; ribbon width corresponds to communication strength. **(D)** Key ligand–receptor pairs from the sender (source) perspective. Bubble plot expanded by “ligand–receptor pair × sender → receiver cell group”; color indicates communication probability, and dot size indicates significance (large black dots: p < 0.01). Major axes include MIF–(CD74+CD44), LGALS9–CD45/CD44, IL16–CD4, and ANXA1–FPR1. **(E)** Key ligand–receptor pairs from the receiver (target) perspective. Same as **(D)**, but visualized from the receiver viewpoint to highlight signaling axes enriched when different cell groups act as targets. **(F)** Reactome enrichment analysis of genes involved in significant cell–cell communications.

The overall communication landscape reveals extensive interactions among major immune cells, with TGFBI^+^ monocytes exhibiting the densest and strongest weighted edges with B cells, plasma cells, and neutrophils, showcasing a clear hub feature (**Figures 8A, B**). Representative interaction networks further summarize high-confidence interactions, suggesting that signals from B cells and TGFBI^+^ monocytes interconnect via multiple pathways and form significant communication loops with neutrophils (**Figure 8C**). At the ligand–receptor level, axes such as MIF–(CD74+CD44), LGALS9–CD45/CD44, IL16–CD4, and ANXA1–FPR1 exhibit higher communication probabilities, with multiple pathways pointing to neutrophils as the receptor end, indicating that neutrophils may serve as critical convergence points for inflammatory signals (**Figures 8D, E**). Reactome enrichment analysis of significantly communicated gene sets reveals positive enrichment entries mainly involving translation/nuclear ribosomes, RNA processing, and immune-related processes, while entries related to the mitochondrial respiratory chain show negative NES, indicating metabolic reprogramming accompanying immune activation (**Figure 8F**). Taken together, the concordance among monocyte expansion, elevated glycolysis-associated activity, monocyte-enriched TGFBI expression, and monocyte-centered communication supported the subsequent monocyte-focused interpretation.

### qRT-PCR validation of candidate genes in the CLP model

**Figure 9:**
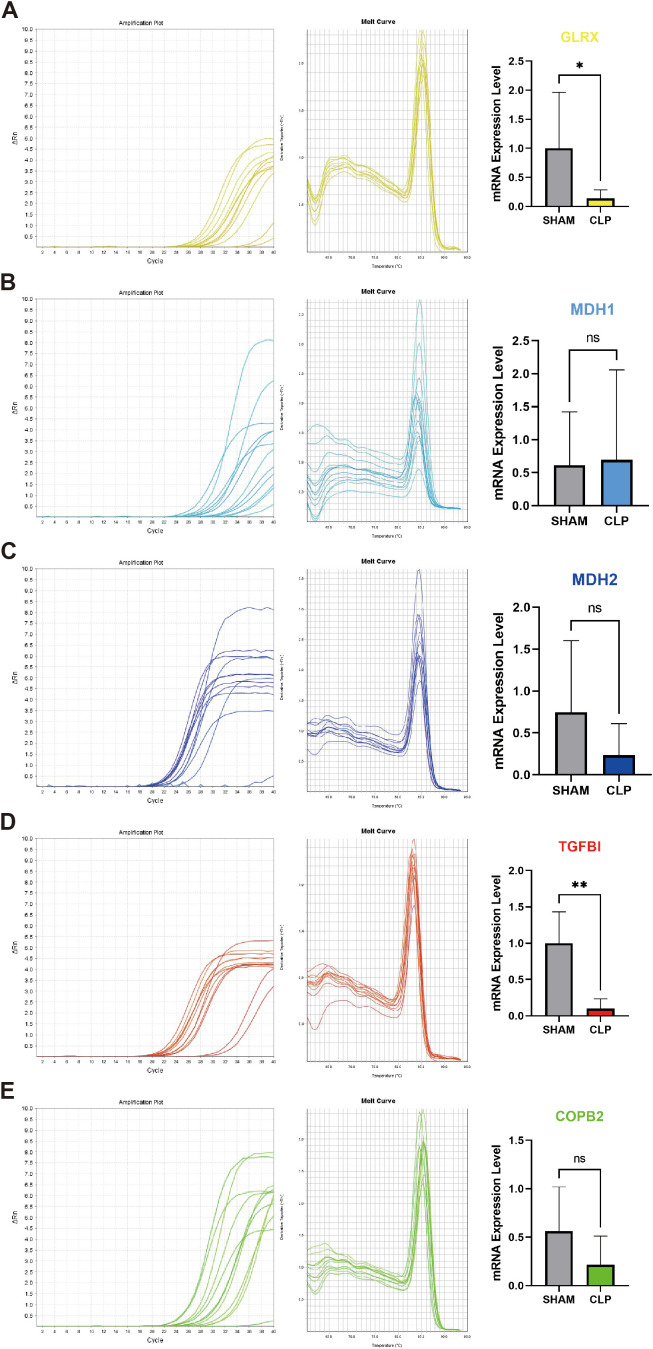
qRT-PCR validation of candidate hub genes in CLP mouse peripheral blood leukocytes. **(A–E)** mRNA expression levels of GLRX, MDH1, MDH2, TGFBI, and COPB2 in peripheral blood leukocyte pellets from CLP mice and control mice.

**Figure 10:**
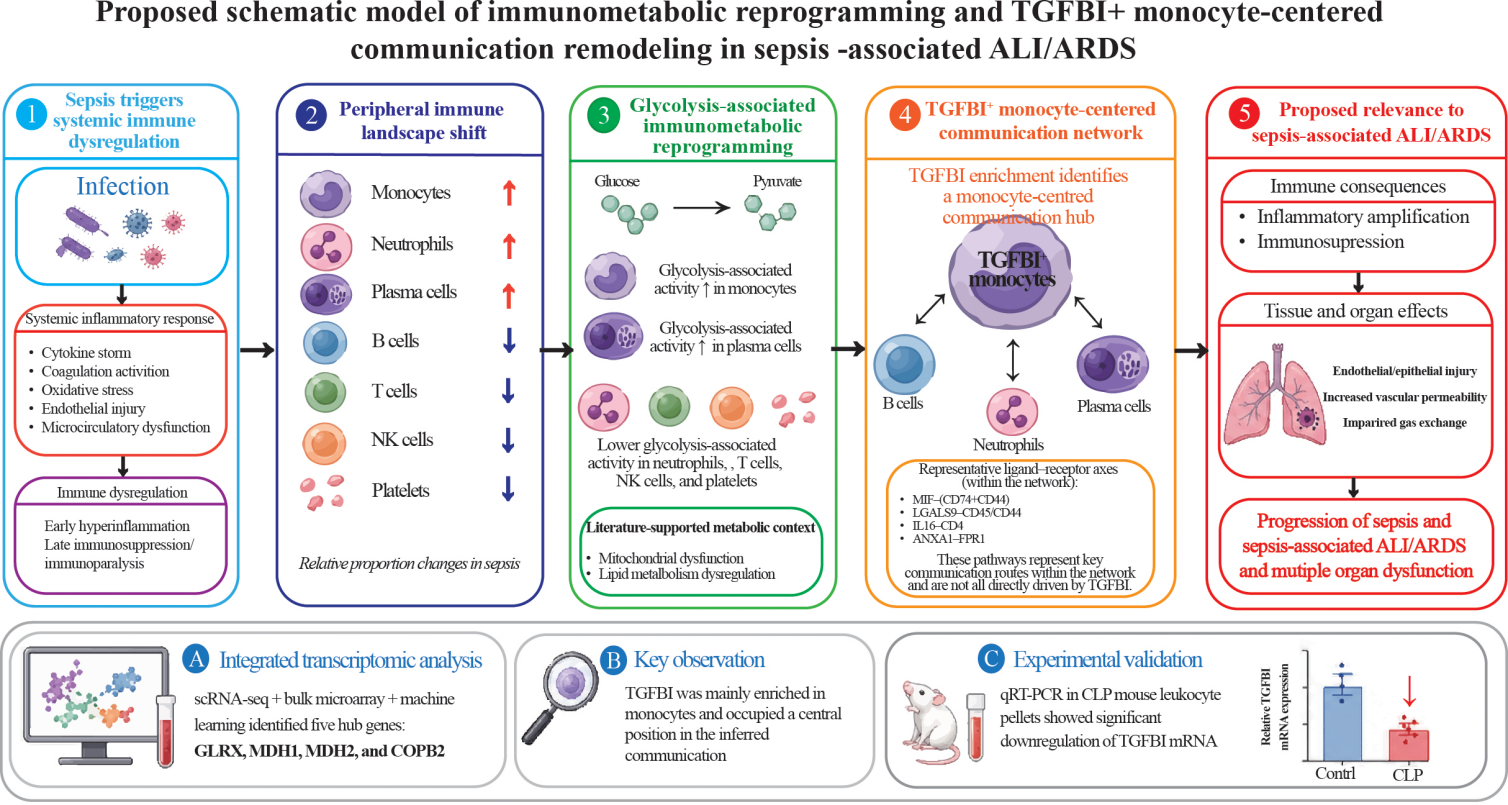
Proposed schematic model of immunometabolic reprogramming and monocyte-centered communication remodeling in sepsis-associated ALI/ARDS. Sepsis induces systemic immune dysregulation and remodeling of the peripheral immune landscape, characterized by relative expansion of monocytes, neutrophils, and plasma cells, together with reduction of B-cell, T-cell, NK-cell, and platelet populations. At the metabolic level, glycolysis-associated activity is increased, particularly in monocytes and plasma cells, while relatively reduced in neutrophils and lymphocyte populations, reflecting cell-type-specific immunometabolic reprogramming. Within this remodeled immune context, TGFBI^+^ monocytes occupy a central position in the inferred intercellular communication network and interact with B cells, plasma cells, and neutrophils through representative ligand–receptor signaling axes (e.g., MIF–CD74/CD44, LGALS9–CD45/CD44, IL16–CD4, and ANXA1–FPR1). These interactions may contribute to amplification of inflammatory signaling and immune remodeling. At the tissue level, these alterations may be associated with endothelial and epithelial injury, increased vascular permeability, impaired gas exchange, and ultimately contribute to inflammatory amplification, immune dysfunction, and progression to sepsis-associated ALI/ARDS. This schematic represents a conceptual summary based on integrated transcriptomic analysis and available literature, and does not imply direct causal relationships.

To validate the five candidate genes identified, we performed qRT-PCR on peripheral blood leukocyte pellets isolated from mice. The results revealed a significant difference in TGFBI between the CLP group and the control group, while MDH1 showed a difference but with an opposite trend. The remaining genes did not reach statistical significance (**Figure 9**). Overall, qRT-PCR validation supported the reproducible between-group difference of TGFBI in an independent mouse cohort, suggesting that TGFBI may represent a relatively robust candidate for further validation. The genes that could not be replicated may be affected by sample heterogeneity, tissue origin differences, or detection window periods, and further validation is needed in larger sample sizes and different tissues/time points. An integrated schematic summary of these findings is provided (**Figure 10**).

## Discussion

This study integrates peripheral blood single-cell and microarray expression data to systematically analyze the immune cell composition, metabolic status, and intercellular communication in sepsis, and identifies five key genes with potential diagnostic value through multi-algorithm screening: GLRX, MDH1, MDH2, TGFBI, and COPB2. Overall, the findings indicate: 1. Monocytes and neutrophils are relatively increased, while the proportions of lymphocytes and platelets are decreased in sepsis; 2. Glycolysis-related activities, represented by HALLMARK_GLYCOLYSIS, are elevated in both the overall population and monocytes/plasma cells, and a set of metabolically associated genes upregulated in alignment with this activity is identified; 3. The intersection of LASSO, random forest, and Boruta algorithms determines the aforementioned five candidate genes, with AUC demonstrating high discriminative power for single-gene prediction by GLRX, TGFBI, MDH1/MDH2, and COPB2; 4. TGFBI is predominantly expressed in monocytes and resides at the core of high-intensity communication networks involving B cells, plasma cells, and neutrophils, with axes such as MIF–(CD74+CD44), LGALS9–CD44/CD45, and ANXA1–FPR1 being particularly prominent. These findings provide single-cell-resolution evidence for the “metabolic reprogramming—myeloid dominance—cross-cellular communication amplification” paradigm of sepsis-induced peripheral immune landscape. Importantly, the monocyte-centered interpretation in the present study was data-driven rather than predefined. Among the major peripheral immune-cell populations, monocytes showed both relative expansion and the most consistent increase in glycolysis-associated activity, while score_UP cells were predominantly localized to monocyte-rich regions. In addition, TGFBI expression was mainly enriched in monocytes, and TGFBI+ monocytes occupied a central position in the inferred communication network. Although plasma cells also displayed increased glycolysis-associated activity, the convergence of compositional shift, metabolic enrichment, and communication centrality made monocytes the most coherent cell population for prioritization in the present analysis.

In our single-cell cohort, an increase in the ratio of monocytes to neutrophils and a decrease in lymphocytes and platelets suggested a typical immunophenotype of “innate immune bias + suppression of adaptive immunity + coagulation dysregulation” in sepsis: infection and systemic inflammation drive stress myelopoiesis, amplifying and dominating peripheral immunity with neutrophils and inflammatory monocytes. However, increased numbers do not necessarily equate to normal function (21), and sepsis is often accompanied by impaired antigen presentation by monocytes and imbalance in chemotaxis/killing by neutrophils (8), resulting in the coexistence of “high inflammation and immunoparalysis”. Sun’s study found that the immune function of several subtypes of monocytes was significantly suppressed in sepsis patients (22). In contrast, peripheral lymphocyte reduction is an established marker of sepsis (23), possibly due to alterations in apoptosis and recycling, thereby weakening pathogen clearance and the establishment of immune memory, and being associated with poor prognosis (24). Reduced platelets reflect excessive activation of the immune-coagulation axis: platelets and neutrophils aggregate and promote the formation of NETs and microthrombi (25, 26), participating in pathogen control while exacerbating microcirculatory dysfunction and organ damage, and simultaneously leading to a decrease in count due to consumptive coagulation.

The activity of glycolysis, characterized by the HALLMARK_GLYCOLYSIS marker, is notably elevated in general, particularly in monocytes and plasma cells, indicating a prototypical immunometabolic reprogramming in sepsis. This shift towards aerobic glycolysis, driven by acute inflammation and hypoxia-like signals in myeloid cells, allows for rapid energy production and provides substrates for biosynthesis, such as lipids and nucleotides (15). Ning et al.’s study reveals that in sepsis, the activation of Hypoxia-Inducible Factor-1α (HIF-1α) is triggered by NAD+ depletion, with high CD38-positive monocytes displaying an overactive glycolysis (19). Accumulation of lactate, acting as a signaling molecule and a donor for histone lactylation (Kla), may amplify inflammatory transcriptional programs and mold subsequent “immunosuppressive/repair” phenotypes. Similarly, Zhang et al. suggest the existence of an “endogenous lactate clock” in M1 macrophages under bacterial attack, which opens gene expression pathways for maintaining homeostasis within the organism (17). Plasma cells, which require a stronger reliance on glycolysis and secretion pathways to meet the high demand for antibody secretion, are more dependent on glycolysis and secretion pathways (27).

Based on the consistent intersection obtained by three machine learning methods, LASSO, random forest, and Boruta, we identified five candidate genes linked to glycolysis-associated metabolic remodeling in sepsis, and single-gene ROC analysis showed that GLRX, TGFBI, MDH1/MDH2, and COPB2 have high discriminative power. From a mechanistic perspective, GLRX reflects the oxidative-reductive homeostasis of glutathione/glutathione disulfide. Reem et al.’s research found that the absence of GLRX increased the expression of SLC7A11 protein and led to a significant increase in GSH content (28). TGFBI may link extracellular-matrix remodeling to changes in the inflammatory microenvironment. Laura et al. suggested that TGFBI may contribute to the immunosuppressive microenvironment in the intraepithelial carcinoma of the uterine tube mucosa, which persists in advanced high-grade ovarian carcinoma (29). MDH1/MDH2 indicates the maintenance of the malate shuttle for NAD (H) balance and the coupling of aerobic glycolysis (30). COPB2 bears the transport pressure of vesicles under high secretory conditions. Li’s research found that COPB2 was highly expressed in bladder cancer, promoting cell proliferation, migration, and glycolytic activity *in vitro* and *in vivo* (31). These axes are highly consistent with the enhanced glycolysis-associated remodeling of myeloid and plasma-cell compartments identified upstream. Methodologically, the intersection of three machine learning algorithms can reduce the contingency brought by single algorithms or data noise. These genes may serve as candidate molecular markers for early identification and risk stratification in sepsis, pending external validation.

Our results indicate that TGFBI is predominantly expressed by monocytes and is situated at the center of a high-intensity communication network with B cells, plasma cells, and neutrophils, suggesting that it may function as an “immuno-matrix” hub molecule in sepsis, coordinating inflammatory and effector responses. In this study, the monocyte-centered interpretation was data-driven, as monocytes showed the strongest convergence of glycolysis-associated activity, TGFBI enrichment, and communication centrality, whereas neutrophils were retained as major interacting cell populations in the inferred inflammatory network. TGFBI is a secreted extracellular matrix protein containing adhesion motifs such as RGD, which can pair with various integrins (32–34). Chen et al. reported that TGFBI in Schwann cells is located at the forefront of cancer neural invasion and can be induced by transforming growth factor-β signaling, promoting tumor cell migration and is associated with poor prognosis. Given its central position in the inferred communication network, monocyte-enriched TGFBI may participate in the coordination of neutrophil- and B/plasma-cell–related responses; however, these functional effects require experimental validation. The centrality of TGFBI in the communication network implies that when monocyte metabolism and inflammatory programs are activated, TGFBI may translate local changes in the extracellular matrix of the inflammatory microenvironment into signal amplification across multiple cell types, thereby linking the observed “myeloid bias + high plasma cell secretion” phenotype. At present, our data do not establish TGFBI as a direct driver of immune dysfunction in sepsis. Rather, TGFBI should be interpreted primarily as a monocyte-enriched, extracellular-matrix-related communication marker that may reflect a broader inflammatory and tissue-remodeling state. Because the communication network was inferred from transcriptomic data and no perturbation experiments were performed, the observed TGFBI-centered pattern remains associative and may represent either an active mediator or a context-dependent readout of coordinated immune remodeling. Likewise, although sepsis involves both hyperinflammatory and immunosuppressive phases, the present study did not include longitudinal or stage-stratified analyses; therefore, we cannot determine whether TGFBI is specifically linked to one phase, spans both phases, or is merely correlated with these transitions. Importantly, TGFBI should not be equated directly with TGF-β1 itself, although it is a TGF-β-inducible extracellular-matrix-related molecule. In sepsis, TGF-β-associated signaling is likely stage-dependent, with potential anti-inflammatory and tissue-protective effects during early hyperinflammation but possible contributions to immunosuppression or immunoparalysis during later phases (35). Because the present study did not directly quantify TGF-β1 activity or perform stage-stratified analyses, the observed TGFBI-centered communication pattern should be interpreted cautiously as a context-dependent association rather than as a direct readout of canonical TGF-β1 signaling. From a translational perspective, however, TGF-β-related signaling in sepsis should still be interpreted cautiously. Representative TGF-β-targeting strategies, including small-molecule TGF-β receptor inhibitors and neutralizing antibodies such as fresolimumab, have mainly been explored in cancer and fibrotic diseases rather than in sepsis (36), and their relevance to sepsis remains largely exploratory. Although some preclinical observations suggest that modulation of TGF-β-associated signaling may help restore immune function during immunosuppressive states, systemic inhibition may also carry the risk of aggravating inflammatory injury. Therefore, TGF-β1 is currently better regarded as a biomarker and mechanistic mediator of sepsis-associated immune dysregulation than as a safe or validated therapeutic target in clinical practice. Future studies may need to focus on more refined approaches, such as stage-specific or cell-type-specific modulation, rather than indiscriminate systemic blockade of TGF-β signaling.

To enhance the credibility of bioinformatics screening results, we further performed qRT-PCR validation in peripheral blood leukocytes of CLP sepsis mice. The results showed that TGFBI exhibited significant differences between the CLP group and the control group, suggesting its certain repeatability in independent biological samples; however, the differences of the other candidate genes did not reach statistical significance, and the change trend of MDH1 was not consistent with the transcriptomic results. This partial concordance should therefore be interpreted cautiously, as the mouse CLP leukocyte-pellet model was used here only as a preliminary orthogonal validation rather than as a one-to-one replication of the human peripheral blood transcriptomic findings. Several factors may account for the limited agreement between computational prediction and experimental validation, including cross-species differences, differences in sample level and cellular composition (human bulk blood/single-cell data versus mouse leukocyte pellets), tissue specificity, and temporal dynamics of gene expression during sepsis progression. In addition, the human single-cell cohort represented late sepsis, whereas the CLP model reflects an experimentally defined acute time window, which may further contribute to directional inconsistency for genes such as MDH1. Therefore, TGFBI can be prioritized as a candidate for subsequent mechanism research and validation, while other genes still need to be further confirmed in a larger sample size, more tissue sources, and multiple time points. To strengthen the conceptual framework of the present study, we incorporated a schematic model summarizing the proposed relationship between peripheral immune-cell remodeling, glycolysis-associated metabolic reprogramming, and monocyte-centered TGFBI communication patterns in sepsis-associated ALI/ARDS.

The strength of this study lies in adopting a multi-cohort integration design of “single-cell (GSE175453) + microarray (GSE100159),” followed by rigorous quality control (QC) and Harmony batch correction, to comprehensively depict the sepsis immune landscape from three dimensions: cellular composition, metabolic status, and cellular communication. Methodologically, the study integrates AUCell scoring, differential/enrichment analysis, and a cross-filtering approach of LASSO-random forest-Boruta to evaluate discriminative power. Biologically, our results support an association between glycolysis-associated programs, myeloid/plasma-cell remodeling, and a TGFBI-centered communication pattern in sepsis, thereby linking molecular alterations to potential functional phenotypes. At the translational level, the identified candidate genes exhibit high single-gene AUC, laying the foundation for future cost-effective detection. Based on the multi-cohort integration of single-cell and microarray data, this study observed a combined feature of innate immune bias and adaptive immune suppression in peripheral immune landscapes of sepsis patients: a relative increase in monocytes and neutrophils, coupled with a decline in lymphocyte and platelet ratios. This pattern aligns with systemic inflammation/stress-induced myelopoiesis, lymphocyte apoptosis, and immune recirculation defects in early sepsis, suggesting that “myeloid upregulation—lymphocyte suppression—coagulation consumption” may constitute the fundamental basis of peripheral immune imbalance. It must be emphasized that this study is observational, and its conclusions primarily reflect associations rather than causality, requiring validation in larger samples and longitudinal follow-up to assess temporal and prognostic significance. Metabolically, we employed AUCell scoring using the MSigDB HALLMARK_GLYCOLYSIS gene set to assess glycolytic metabolic activity in single cells, serving as a proxy indicator. This revealed significantly elevated scores in both overall and monocyte/plasma-cell populations, leading to the identification of a set of metabolically related genes (GLRX, MDH1/MDH2, TGFBI, COPB2) that correlate positively with this activity. These genes point to the axes of redox homeostasis, malate-aspartate shuttle, and secretory/vesicular transport, consistent with our observations on cellular composition and function. However, the transcriptional layer of glycolytic activity does not equate to the proteomic level of “histone lactylation (Kla)”; the former is more likely to be an upstream or parallel metabolic background for the latter. Therefore, further validation should be conducted by integrating metabolomics and proteomic detection of Kla. By combining LASSO, random forest, and Boruta methods, we obtained consistent intersections and identified five candidate genes. Single-gene ROC analysis showed that GLRX, TGFBI, MDH1/MDH2, and COPB2 exhibited high discriminative power. The intersection of the three methods is advantageous in reducing false positives; however, it may also miss true signals identified by single methods alone. Therefore, further construction of multi-center external validation will better reflect their clinical applicability and transferability. We observed that TGFBI-positive monocytes are located at the center of high-intensity communication circuits with B cells, plasma cells, and neutrophils, suggesting that they may act as a “immune–stromal” hub, influencing adhesion/migration, antibody secretion, and inflammation amplification via integrins and other ligand-receptor axes. Note that CellChat infers potential ligand-receptor “communications” based on transcriptional expression, and the network strength and centrality do not directly correlate with the necessity or flux at the proteomic level. Subsequent causal validation should integrate spatialomics/imaging co-localization and neutralization/blocking experiments. Although the CLP model provided preliminary biological support for TGFBI, the translational relevance of the proposed biomarkers remains limited in the absence of validation in an independent human sepsis cohort or ICU-based patient samples. Therefore, the current findings should be interpreted as a biomarker-prioritization framework rather than as a clinically validated diagnostic signature. Future studies should test these candidates in multicenter human cohorts with longitudinal sampling and outcome annotation to determine their diagnostic robustness, stage specificity, and potential utility for risk stratification. An additional limitation is the heterogeneity of the public datasets used in this study. GSE175453 represents a late-sepsis single-cell cohort, whereas GSE100159 is a whole-blood microarray cohort, and the two datasets differ in disease stage, sample composition, and transcriptomic platform. Therefore, our results should be interpreted as identifying shared glycolysis-associated signatures across heterogeneous sepsis-related datasets rather than defining a single homogeneous sepsis subtype. Accordingly, the extrapolation of conclusions requires confirmation in larger clinical cohorts.

In summary, we have delineated the characteristics of sepsis peripheral blood “myeloid bias - metabolic reprogramming - amplification of intercellular communication” at the single-cell resolution level, identifying a panel of metabolically relevant genes with discriminative potential. Based on this, we propose that future endeavors should concentrate on multicenter prospective validation and functional experiments targeting metabolic and communication axes, aiming to facilitate the translational loop from molecular phenotyping to clinical stratification and intervention targets.

## Data Availability

The datasets presented in this study can be found in online repositories. The names of the repository/repositories and accession number(s) can be found in the article/supplementary material.
